# Family-Level Diversity of Hymenopteran Parasitoid Communities in Agricultural Drainage Ditches and Implications for Biological Control

**DOI:** 10.3390/insects16030246

**Published:** 2025-02-27

**Authors:** Shane Daniel Windsor, Alireza Shokoohi, Robert Salerno, William Lamp

**Affiliations:** 1 Department of Entomology, University of Maryland, College Park, MD 20742, USA; ashokoohi@umass.edu (A.S.); rsalerno@umd.edu (R.S.); lamp@umd.edu (W.L.); 2 UMass Extension, University of Massachusetts Amherst, Amherst, MA 01003, USA

**Keywords:** farmscape, conservation biological control, parasitoid wasps, biodiversity, sustainable agriculture

## Abstract

Agricultural drainage ditches contain a variety of non-crop vegetation, including potential sources of alternate hosts and food for hymenopteran parasitoids that provide conservation biological control on adjacent farm fields. To assess the patterns of family-level diversity of hymenopteran parasitoids, we surveyed ditch and adjacent crop habitats during June, July, and August 2021–2023, using yellow sticky traps over one week. We sampled two agricultural drainage ditches on each of five farms on the Delmarva Peninsula, eastern USA. We collected 36,725 specimens and identified 29 families across 738 sticky traps. Parasitoid diversity was greater in agricultural ditches than in adjacent fields. While parasitoid family diversity and abundance varied across the farms, ditches within a farm were similar. Within crop fields, diversity was greater at 1.5 m from agricultural ditches than at 9.1 m from the ditches. For several well-sampled families, greater abundance on one farm relative to others extended to both ditches and adjacent crops. Our findings indicate that agricultural drainage ditches serve as an existing beneficial semi-natural habitat for parasitoids on farms. Further research into ditch management practices may reveal methods of enhancing parasitoid abundance and conservation biological control while requiring relatively little investment from farm managers.

## Simple Summary

Conservation biological control consists of practices that often use semi-natural habitats to increase beneficial natural enemies on farms. While agricultural drainage ditches are designed to control the water table on farm fields, natural enemies may exploit their diverse, natural vegetation as a source of food, hosts, and/or shelter adjacent to cropland. Among those natural enemies are parasitoid wasps, a diverse group of insects that develop and kill invertebrate hosts, including potential pests in crop fields. These natural enemies serve as the foundation for the sustainable management of arthropod pests in agroecosystems. To evaluate the value of drainage ditches for supporting natural enemies that are important to conservation biological control on farms, we surveyed parasitoid wasps using sticky traps in ditches and adjacent crop fields on five different farms. Wasps were more diverse within agricultural ditches than adjacent crops and diversity within the crops was greater in samples taken closer to agricultural ditches. Families differed in abundance between ditches and crops of different farms. Our results indicate that parasitoid wasps inhabit agricultural ditches for food or shelter, laying the groundwork for their development for conservation biological control of pests.

## 1. Introduction

Approximately 44% of land cover in the United States has been converted from natural habitat to agricultural landscapes since the sixteenth century [1,2]. Although many ecosystem functions have been lost through this conversion, agroecosystems continue to provide important cultural, supporting, and regulating ecosystem services, including landscape aesthetics, decomposition, pollination, and biological control [3]. However, current agricultural management practices such as excess fertilization, pesticide use, tillage, and crop monocultures impose negative consequences for ecosystem health and biodiversity [4]. The intensity of ecosystem services provided by agroecosystems has diminished as a result [5,6]. Balancing the imperative of increasing crop and livestock production while simultaneously mitigating environmental degradation is one of the paramount challenges of our time [7]. Ecological intensification through the management of service-providing organisms has the potential to reduce environmental disturbance and our reliance on external inputs while maintaining or increasing food, fiber, and fuel production [8].

The incorporation of diverse plant communities within and around agricultural landscapes can positively influence pollinator and natural enemy populations by providing alternative resources such as food and shelter, supporting their survival and reproduction [9,10]. Increasing populations of beneficial insects may reciprocally increase ecosystem service provisioning, benefiting agricultural production [4,10]. Preexisting semi-natural habitats within agroecosystems already provide these services, reducing the need for external inputs like pesticides to control pests [11,12].

Agricultural drainage ditches are one such example of preexisting semi-natural habitats. In low-lying areas prone to flooding, such as the coastal plain of the Delmarva Peninsula, excess water is diverted out of crop fields through agricultural drainage ditches [13]. Surface runoff and subsurface water flow into the channel, particularly when high volumes of rainfall cause flooding and raise water tables. The slope of the channel carries water away from agricultural fields, allowing more water to continue draining from the field. These ditches are highly variable in terms of many of their characteristics, including their physical shape and dimensions, water flow, and ecological characteristics, including plant, invertebrate, and vertebrate diversity. Agricultural drainage ditches are less intensely managed than crop fields. To prevent woody perennial plants from becoming established, ditches are often mowed only once a year in the fall. Weed problems may be treated manually or with herbicide spot treatments, but unproblematic plants are often left to grow in the ditches, resulting in greater plant and floral diversity on farms. This provides a wealth and diversity of resources, including food and shelter for beneficial arthropods such as parasitoids, giving rise to ecological benefits to agriculture that supplement the hydrological purpose of drainage ditches [14,15]. Here, we evaluate the potential of agricultural drainage ditches to support agents of conservation biological control of crop pests, with a focus on parasitoids in the order Hymenoptera.

Parasitoidism, a form of parasitism that involves laying eggs on or inside a host (egg, larvae, pupa, and, less commonly, adult), invariably killing the host as they develop and emerge, is the most prevalent strategy among Hymenoptera. The high diversity and host specificity of hymenopteran parasitoids has led to them being commonly employed in classical and augmentative biological control strategies, in which organisms are introduced into a system to control pest species [16]. While these strategies can be successful, with fewer adverse effects compared to chemical alternatives, they do nevertheless require external inputs, which may pose environmental risks and ongoing monetary expenses [17,18]. In contrast, conservation biological control describes the manipulation of environmental factors in a system to maximize the efficacy of existing natural enemy populations, such as by increasing the available food and shelter for parasitoid wasps to increase their diversity. Greater diversity among parasitoid wasps has been linked to more consistent biological control on farms [19].

Because parasitic Hymenoptera are such a diverse group, their life cycle is difficult to generalize broadly within each family. Many parasitoids, such as mymarids, overwinter within their hosts as late-stage larval instars, while other parasitoids overwinter as adults [20,21]. Larger families contain both univoltine and multivoltine life cycles, making it difficult to identify the generations per year of a given specimen [22]. Host selection involves utilizing scent indicators from either the host itself or from other organisms, such as plants or fungi associated with them [23]. While larvae gain sustenance from hosts, adult parasitoids often make use of nectar as a food source.

Like many other natural enemies, hymenopteran parasitoids in agricultural systems can benefit from semi-natural habitats as a source of alternate hosts or floral nectar [24]. Access to the latter has been shown to lead to increased rates of parasitoidism among hymenopterans [25], while the former can play important roles in a parasitoid’s overwintering life cycle. Agricultural ditches can provide a source of non-crop vegetation, including native nectar-rich plants and shrubbery favored by lepidopteran hosts [26]. This suggests that agricultural drainage ditches may support parasitoid wasps in agroecosystems and act as a source of natural enemies, which can enhance biological control in nearby crops.

Previous research has found that other semi-natural habitat types benefit nearby hymenopteran parasitoid populations [11,27,28]. Purposefully selected, artificially created grass buffers in the same region have also been demonstrated to benefit parasitoids in adjacent crops [29]. To better understand the influence of agricultural drainage ditches on parasitoid wasp communities on farms, we aimed to (a) identify major parasitoid families within agricultural ditches and patterns across communities, and (b) analyze the relationship between parasitoid communities within agricultural ditches and those in adjacent fields to determine the relationship between the two habitats. We expected to find a greater abundance and diversity of parasitoid wasps in ditches, with abundance and diversity decreasing as distance from the ditch increases. 

## 2. Materials and Methods

### 2.1. Study Area

**Figure 1:**
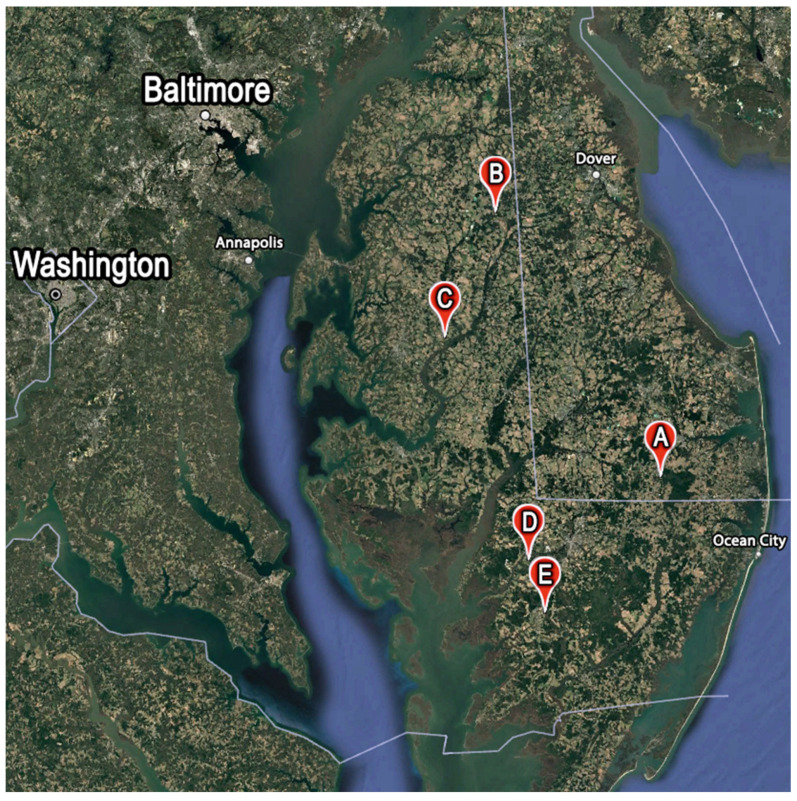
Map view of the five different farms (Farm A–Farm E) that were sampled on the Delmarva Peninsula. The letters of each label correspond to the location of the sampled ditches at each of the farms. Created with Google Earth, Map Imagery Landsat / Copernicus, Map Data SIO, NOAA, U.S. Navy, NGA, GEBCO.

Five farms on the Delmarva Peninsula, USA, were selected as sites for this study to represent the range of farm types found in the region and were sampled from 2021 to 2023 (Figure 1). Farm A was a conventional poultry farm producing continuous no-till corn utilizing poultry litter as fertilizer, with moderately deep ditches that often had shallow standing water and diverse vegetation. Farm B was a conventional poultry farm producing corn/soybean in rotation with winter rye cover cropping, and ditches were moderately deep with standing water. Farm C was a conventional grain farm producing in a corn/soybean rotation with a deep ditch featuring a permanent water flow from a headwater stream and lush, tall vegetation throughout the year (Table S1). Farm D was an experimental, conventional farm managed by the University of Maryland Eastern Shore with shallow, often dry ditches that mostly contained short grasses with little plant diversity. Farm E was an organic farm utilizing rye harvested early in summer followed by tilled soybeans fertilized with poultry litter; the ditches possessed diverse vegetation. Approximately half of the Delmarva Peninsula is used for agriculture, including much of the surroundings of the sampled farms [30]. The specifics of the crop rotations may be found in Table 1. To further characterize the farms, two soil samples were collected on each farm, one for each ditch, and tested for physical and chemical composition, while the physical characteristics of ditches (such as the total length and width) were measured at each transect for each farm. The measured characteristics of ditches on the sampled farms varied, with all the measurements differing between the farms (Kruskal, *p* < 0.01) (Table 2). Soil samples found significantly higher levels of phosphorus on farm A compared to other farms (ANOVA, *p* < 0.001). Aside from the greater phosphorus levels on farm A, other soil tests did not find significantly different results across farms.

### 2.2. Sampling

**Figure 2:**
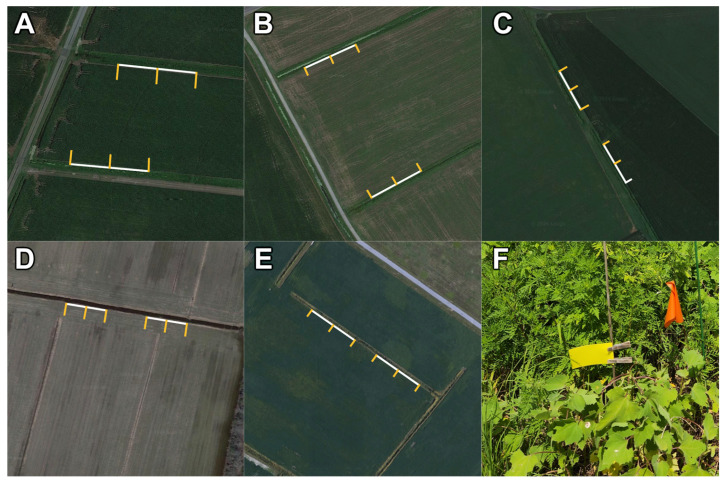
Satellite imagery of ditches and transects sampled for each of the five farms studied (farms (**A**–**E**)) and a photograph of a sticky trap set up in the field (subfigure (**F**)). Drainage ditch segments (white) on each farm were defined as bordering the same field either opposing each other (farms (**A**,**B**)) or adjacent to each other with 30.4 m between ditch segments (farms (**C**–**E**)). Sampling was conducted along transects perpendicular to the longer segments (yellow), which were located 30.4 m apart at all sites except farm (**D**), where they were located 15.2 m apart. Map Imagery ©2024 Google.

Two ditch segments bordering the same field were chosen on each farm, and three transects were defined beginning at each ditch segment and extending perpendicular to the ditch into the field. Samples were deployed at three locations along each transect: at the top edge of the slope of the ditch, 1.5 m into the adjoining field, and 9.1 m into the field (Figure 2). Double-sided yellow 7.6 × 12.7 cm sticky traps (PHEROCON^®^ Predator 3″ × 5″ Traps, Trécé Incorporated: Adair, OK, USA) were attached to dowel rods with clothespins with the bottom edge of the trap either 41 cm above the ground (when surrounded by taller vegetation, such as in a cornfield or some larger ditches) or just above the vegetation line (around shorter vegetation, such as in young soybean fields). Each sticky trap was laminated in clear plastic with a paper label and placed in a freezer for later identification.

The sampling periods lasted 7 days and were completed once per month in June, July, and August for each of three years (Table S1). In total, 756 sticky cards were deployed and 738 were successfully collected and processed, with the rest missing due to weather or other circumstances. Specimens were analyzed under a magnifying scope and identified to family level using the dichotomous key found in Goulet and Huber [31]. Due to a lack of up-to-date keys, the families Aphelinidae and Pteromalidae were identified using older definitions, and each should be understood to refer to Aphelinidae and Pteromalidae sensu lato. Specimen counts of each family for each sticky trap were recorded.

### 2.3. Analysis

All the analyses were performed using R Statistical Software (v4.4.2; R Core Team 2024). As family abundance was non-normal, comparisons of means were conducted using Dunn Kruskal–Wallis multiple comparison tests with Holm-adjusted *p*-values (with α = 0.05) using the dunn.test function from the dunn.test package (version 1.3.6) [32]. Differences in community composition were evaluated through both permutational multivariate analysis of variance (PERMANOVA) based on Bray–Curtis dissimilarity using the adonis2 command from the vegan package (version 2.6-6) and pairwise PERMANOVA based on Bray–Curtis dissimilarity using the pairwise.adonis2 command from the pairwiseAdonis package (version 0.4.1) [33,34]. For replicability, the seed was set to 42 using the set.seed() function before running PERMANOVA or pairwise PERMANOVA tests. Rényi diversity profiles were used to compare diversity between the locations and farms sampled, as they allow for direct comparison of common diversity metrics in addition to being generally accepted as one of the more useful methods [35]. Rényi diversity profiles were obtained through the renyicomp function from the BiodiversityR package (version 2.16.1), using 1000 permutations with the smallest sample size among compared categories to determine margins of error and testing at α = 0, 0.25, 0.5, 1, 2, 4, 8, and infinity [36].

## 3. Results

### 3.1. Agricultural Drainage Ditch Characteristics and Parasitoid Communities Within Ditches

In total, 16,022 specimens from 29 families were collected within the semi-natural habitat of drainage ditches, and 36,725 specimens were collected overall.

#### 3.1.1. Community Composition and Overall Abundance

Differences between communities across farms were significant, with farms accounting for 13% of the total variance in communities for each sample (PERMANOVA, *p* = 0.001). Differences between months and years were also significant, accounting for 6% and 5% of the total variance in communities for each sample (*p* = 0.001) (Table 3). Pairwise PERMANOVA found that all farms had significantly different community compositions from one another (*p* < 0.05), except for farms B and D (*p* = 0.141) (Table S3). Farms B and D also had significantly different dispersions from farm C (PERMDISP, *p* < 0.05), indicating that while farm A and E were clearly different in distribution from farms B, C, and D, it is unclear whether the differences between farm C and farms B and D were the result of variance or truly different distributions. No differences in community composition or overall abundance were found between the two ditches sampled for any of the farms (pairwise PERMANOVA, *p* > 0.05 for all pairs). Additionally, community composition in ditches did not differ between adjacent crop types (*p* = 0.741). Overall abundance in ditches did not significantly differ between June and August but was lower in July. However, overall abundance did not differ between farms (Kruskal, Holm adj. *p* > 0.05 for all).

#### 3.1.2. Common Families in Ditches

Hymenopteran parasitoids from 29 different families were sampled from the non-crop habitat within the agricultural drainage ditches. While mymarid wasps were the most abundant family within ditches, scelionid wasps were the most commonly occurring, present on 99% of all traps in ditches (Table 4). Commonly occurring families (i.e., those present in at least 50% of all sampled sticky cards) include Mymaridae, Trichogrammatidae, Scelionidae, Figitidae, Ceraphronidae, Platygastridae, Aphelinidae, Eulophidae, Encyrtidae, Pteromalidae s.l., and Braconidae.

**Figure 3:**
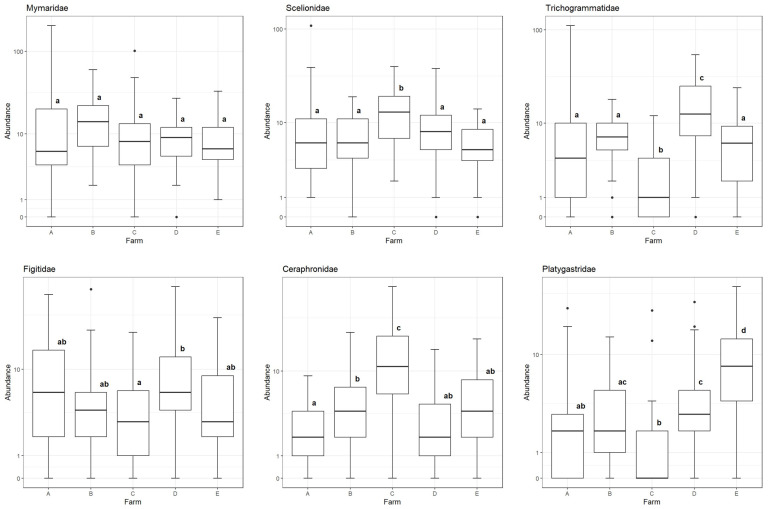
Box plots of sample abundance for the six most common families in agricultural ditches at each farm, using a pseudo-log scale. Plots with a different letter indicate that the mean abundances are different from one another at the α = 0.05 significance level (per Holm-adjusted Dunn tests).

Of the common families, Mymaridae was the only one without significant differences between sample means across different farms (Figure 3). Trichogrammatid wasps were much more abundant within ditches on farm D than within ditches on other farms, and least abundant within the ditches of farm C. In contrast, farm C had greater scelionid and ceraphronid wasp abundance in ditches than on any other farm. Platygastrid wasps were most abundant on farm E.

Between months, the families Mymaridae and Trichogrammatidae were both much more abundant in August than the other two months, while being less abundant in July than June or August (Table 5). The families Aphelinidae and Pteromalidae s.l. were more abundant in June than in July and August. The families Scelionidae, Ceraphronidae, Encyrtidae, Platygastridae, and Braconidae had the same level of average abundance throughout the period sampled.

### 3.2. Relation Between Ditch and Adjacent Crop Habitat

#### 3.2.1. Community Composition

Pairwise PERMANOVA found all three sampled distances (in ditch, 1.5 m into crop, and 9.1 m into crop) to have significantly different distributions from one another (*p* = 0.001 for all pairs), albeit to lesser extents (R^2^ = 0.05 for ditch vs. 1.5 m into crop, 0.09 for ditch vs. 9.1 m into crop, and 0.009 for 1.5 m vs. 9.1 m into crop) (Table S4). PERMDISP found significant differences in dispersion both between the ditch and 1.5 m into the crop and between the ditch and 9.1 m into the crop (*p* = 0.0001) but not between 1.5 and 9.1 m into the crop (*p* = 0.88), which may indicate that the community compositions of the two distances into the crop have differences in distribution that cannot be accounted for solely by variance. These results demonstrate differences between the three sampled distances. Unlike samples taken in nearby ditches, the crop type sampled was a significant factor in the variance of samples taken from within crops (R^2^ = 0.014, *p* = 0.001) (Table S5).

#### 3.2.2. Common Families in Ditches and Adjacent Crops

Of families present in at least 50% of samples, only Mymaridae and Diapriidae did not differ significantly in abundance between samples from ditches and the two distances into the crop (Holm-adj. *p* > 0.12 for both). Trichogrammatidae, Figitidae, Ceraphronidae, Platygastridae, Eulophidae, and Encyrtidae were significantly more abundant within the ditch habitat than either form of crop habitat (Holm-adj. *p* < 0.01) but did not differ in abundance between 1.5 m and 9.1 m into the crop. Scelionidae was also more abundant within the ditch habitat than either of the two distances into the crop, but the two crop habitats differed from one another, with greater abundance 1.5 m into the crop than 9.1 m into the crop (Holm-adj. *p* < 0.01). Braconid wasps were more abundant within the ditch habitat and 1.5 m into the crop than 9.1 m into the crop (Holm-adj. *p* < 0.05), and did not differ significantly in abundance between ditches and 1.5 m into the crop (*p* = 0.61). Samples within fields also varied between farms (Table 6). Farm D had greater trichogrammatid abundance and farm C had greater scelionid and ceraphronid abundance within field locations than the other farms (except farm E). Platygastrids were more abundant in the fields of farm E than any other farms.

#### 3.2.3. Temporal Differences

**Figure 4:**
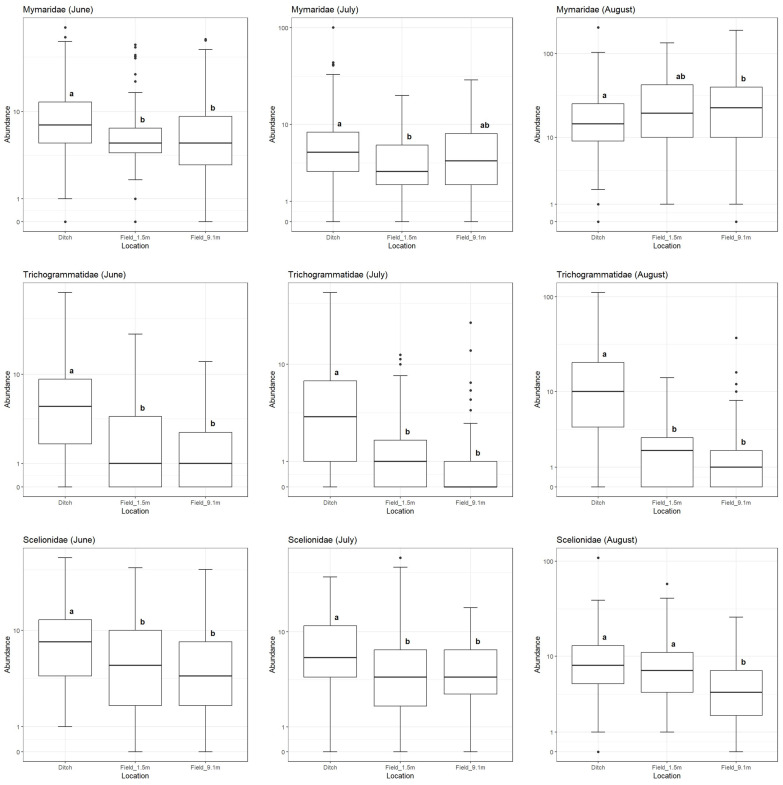
Box plots of sample abundance for the three most common families in this study for each month, using a pseudo-log scale. Plots with a different letter indicate the mean abundances are significantly different from one another at the α = 0.05 significance level (per Holm-adjusted Dunn tests).

Some patterns in the data do not hold true when separated across months throughout the year. Mymarid wasps were more abundant in agricultural ditches than either of the crop locations during the month of June, but more abundant in the crop locations than in agricultural ditches during the month of August (Figure 4). Scelionid wasps had greater abundance in ditch habitats than either of the crop locations during the first two months sampled but had equal abundance in the ditch habitat and the 1.5 m crop location during August (both of which were greater than the 9.1 m crop location). Trichogrammatid wasps, by contrast, demonstrated greater abundance within ditches compared to crop habitat across all the months sampled. Thus, mymarid wasps, and to a lesser extent scelionid wasps, demonstrated movement into the crop during the summer months, while trichogrammatid wasps did not demonstrate movement.

#### 3.2.4. Diversity Profiles

**Figure 5:**
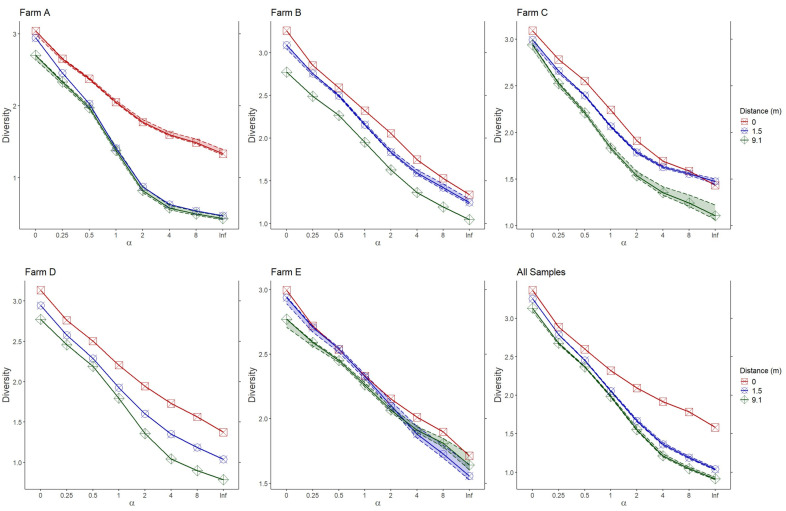
Rényi diversity profiles of each farm and of all samples collectively, comparing samples taken within the ditch (0 m) with varying distances away (1.5 m and 9.1 m). Each graph compares Hill numbers to assess multiple diversity metrics at once (family richness at α = 0, Shannon diversity at α = 1, Simpson index at α = 2, etc.) with the α of the Hill number mapped to the *x*-axis and the value of each diversity metric mapped to the *y*-axis.

Clear differences in diversity existed between samples collected within ditches and adjacent crops at all farms except for farm E, where there was no clear order (Figure 5). The remaining four farms found samples from agricultural ditches to have greater diversity than samples taken 9.1 m into adjacent crops. Farms B, C, and D had greater diversity in crop samples taken 1.5 m from the ditch than those further away. All the farm data showed similar trends when measured collectively.

## 4. Discussion

Conservation biological control relies on the presence of a permanent, diverse, lightly managed habitat to support the growth, reproduction, and survival of beneficial natural enemies [37]. Our study demonstrated that agricultural drainage ditches, common on farms with a high-water table and flat topography, may serve as sources for parasitic Hymenoptera as agents for conservation biological control in adjacent crops. Throughout this three-year study, we collected 36,725 parasitoid wasps from 738 sticky traps on five farms. We discovered high diversity among parasitic Hymenoptera within ditches, and less so in the adjacent crops. Of the 29 families sampled, we found the taxonomic diversity varied among farms, years, and months, but not between ditches within farms. Families differed in their spatial and temporal patterns, but a few families (notably Mymaridae and Scelionidae) gained relative abundance in the crop relative to the ditch as the summer proceeded. Our results suggest that agricultural drainage ditches play a significant role in supporting parasitoid communities in agroecosystems on the Delmarva Peninsula.

Diversity profiles taken across all samples indicated that hymenopteran diversity is greatest in the ditch, with diversity decreasing with distance into the crop field. This pattern resembles those seen in other similar systems where populations of natural enemies are supported by a diverse non-cropped semi-natural habitat [29]. This pattern did not always hold true when profiles were taken across separate farms. While most Hill numbers for farm A support the conclusion that diversity increases with ditch proximity, it is possible that characteristics or management practices of the ditch or adjacent fields have inhibited the benefits of the ditch on diversity compared to others. Farm E did not have greater diversity at any of the sampled locations, which could also relate to its management practices, such as organic farming. Further studies could elucidate the effects of these or other management practices (such as organic farming, changing the time of year mowing takes place, or adding straw) that have been demonstrated to impact terrestrial natural enemies on parasitoid populations within agricultural ditches [38].

Our results may derive from agricultural drainage ditches serving as overwintering sites or sources of alternate hosts and nectar. Multiple studies have demonstrated that other kinds of semi-natural field borders can support natural enemy biodiversity through the provision of overwintering refugia [11,12,27]. Both parasitoids that overwinter inside and outside of hosts can benefit from agricultural drainage ditches, as cultivated land is left fallow or planted with homogenous cover crops providing comparatively low resource availability throughout the winter. During warmer months, a semi-natural habitat can provide floral resources that are not present in nearby monoculture fields, enhancing the rates of parasitism among Hymenoptera in several other studies investigating semi-natural habitat [39,40].

Mymaridae, Trichogrammatidae, and Scelionidae were the most abundant families, which is consistent with studies of grass buffers from the Delmarva Peninsula [29,41]. These studies also found that different plant types within those buffers had an impact on the diversity of several species [29]. The increased abundance of trichogrammatid wasps within ditch vegetation and adjacent crops of farm D, characterized by grassy vegetation, aligns with previous findings indicating that this wasp family shows higher abundances in and near warm-season grass buffers. These buffers, like the vegetation at farm D, are mostly composed of perennial grasses, providing a favorable year-round habitat [29]. Additionally, Trichogrammatidae, Scelionidae, Ceraphronidae, and Platygastridae were all significantly more abundant in the fields of farms, where they were more abundant in the ditches (compared to other farms). These results suggest that parasitoid communities within the two habitat types are connected on each farm.

While there were many differences in community composition between the farms, there were no differences in abundance between them. This is despite the significant differences in the overall sizes of the sampled ditches, as other studies have found that increased size of semi-natural habitats can lead to greater abundances of hosts or nectar sources [42,43]. Additionally, despite close geographical proximity, farms D and E had different community compositions, with farm D having more in common with farm B, which was further away. Farm identity was the greatest predictor of parasitoid community composition, suggesting that other factors, such as plant community composition or land management, serve as primary drivers of parasitoid wasp community dynamics on these farms. In several other studies, plant community composition was identified as an important predictor of natural enemy diversity across habitats. Within agroecosystems, species richness and abundance were negatively correlated with grasses and highly correlated with forb cover [27]. Other studies have found parasitoid diversity and richness to increase in association with areas of higher plant diversity in forest and grassland-type habitats [44,45,46]. In agricultural drainage ditches studied here, the diverse plant taxa may have contributed to microhabitat heterogeneity, influencing parasitoid communities. The variability observed in vegetation structure across ditches may also have contributed to the differences observed in parasitoid communities across the five farms. Several studies have determined that increasing vegetation complexity (e.g., vegetation height, stem count, and leaf pattern) increases alternative prey and nectar resources—factors that have generally been linked to increased parasitoid taxa richness [42,47].

Farm identity was followed closely by sampling month and year in explaining patterns in parasitoid wasp communities, indicating that wasp communities in drainage ditches change over the season. Several families were found in greater numbers in either June or August, while others were found at around the same level of abundance throughout the season. Annual variation in temperature and indirect impacts of climatic fluctuations on communities of other organisms such as plants and herbivores present in ditches have also been shown to influence parasitoid wasp communities [48,49]. On a given farm, families that were common in the ditch were also found frequently in the field. Mymarid wasps were more abundant in ditches than in crops in June but were collected more frequently in crops in August, implying that there may be directional movement of wasps from this family into the field over the summer.

Identification of specimens to species may reveal additional similarities and differences between these communities. However, the overwhelming number of specimens and relative difficulty in identification for many lower-level taxonomic groups alongside some key characteristics requiring removal from sticky traps makes thorough identification of all specimens to the species level too time-intensive for a study of this scale. Nevertheless, species identity is an important characteristic where parasitic Hymenoptera are concerned due to the hyper-diverse and specialized nature of most parasitoid wasps. The lack of species identification is an obstacle to understanding the applicability of these results to conservation biological control. Our results may also have been affected by sampling biases associated with sticky traps. While yellow sticky traps have been noted in comparative studies to be one of the more effective methods of sampling hymenopteran parasitoid populations, there are some biases associated with them [50]. For instance, it should be noted that sticky trap capture rates do not directly represent a sample of the absolute abundance of captured species but rather a product of abundance and activity. Thus, it is likely that our comparisons of parasitoid communities between ditches and crops were affected by differences in plant structure in each habitat. Additionally, capture rates could have been influenced by variations in size, flight behavior, and attraction to yellow sticky cards.

Despite these limitations, yellow sticky traps are effective at capturing parasitic Hymenoptera [50]. Compared to malaise traps, which are also commonly used for sampling small aerial insects, sticky traps can be set up quickly and efficiently at multiple sites in agricultural terrain, such as within a cornfield. Additionally, family-level identification of hymenopteran parasitoids is dependent on morphological features such as wing venation that are just as apparent on sticky traps as on mounted specimens, allowing for quick identification of many samples. Because of this, it is possible to process and store specimens quickly and efficiently on sticky traps. Unlike active sampling methods, such as foliar sweeps, sticky traps also have the benefit of capturing activity throughout the day, minimizing bias associated with activity patterns of species with contrasting diurnal behaviors.

Overall, our results demonstrate the value of agricultural drainage ditches as preexisting non-crop habitats which support communities of parasitoid wasps in agricultural landscapes. Parasitoid diversity was greatest in samples within agricultural ditches compared to within adjacent crops, with diversity decreasing with sampling distance into the field. Several abundant parasitoid families were more prevalent in the ditches and nearby fields of specific farms compared to others. Due to the diversity of parasitoid taxa in their natural histories, agricultural drainage ditches that differ in their characteristics, spread throughout the agricultural landscape of the Delmarva Peninsula, should result in the conservation of highly diverse parasitoid wasp communities. Future research focused on altering drainage ditch management practices could enhance the benefits of these habitats on farms with lower investment of time, money, and labor compared to constructing and maintaining new semi-natural habitat.

## Figures and Tables

**Table 1:** Crops planted near sampled ditches on each farm.

Farm	Crop Type		
	2021	2022	2023
A	Corn	Corn	Corn
B	Corn	Soy	Corn
C	Soy	Corn	Soy
D	Corn	Soy	Corn
E	Soy	Soy	Soy

**Table 2:** Mean (±S.E.) for measured characteristics of agricultural drainage ditches sampled in the study.

Farm	Ditch	Length (m)	Riparian Distance (m)	Width (m)	Depth (m)	Channel Width (m)
A	NO	385	2.0 ± 0.1	4.9 ± 0.2	1.1 ± 0.1	1.9 ± 0.1
	SO	430	0.9 ± 0.1	3.6 ± 0.0	0.5 ± 0.1	1.8 ± 0.0
B	NO	321	2.1 ± 0.1	6.1 ± 0.1	1.2 ± 0.2	1.4 ± 0.1
	SO	400	3.1 ± 0.2	4.7 ± 0.4	0.9 ± 0.1	1.2 ± 0.1
C	NO	786	8.8 ± 0.1	6.1 ± 0.1	0.9 ± 0.1	0.9 ± 0.1
	SO	786	1.3 ± 0.1	5.8 ± 0.2	0.5 ± 0.1	1.2 ± 0.2
D	EA	192	2.9 ± 0.1	3.7 ± 0.1	0.5 ± 0.0	1.5 ± 0.1
	WE	192	2.8 ± 0.1	4.2 ± 0.1	0.4 ± 0.0	1.7 ± 0.2
E	NO	212	0.2 ± 0.1	3.8 ± 0.1	0.6 ± 0.0	1.1 ± 0.1
	SO	218	0.6 ± 0.1	3.8 ± 0.2	0.8 ± 0.0	1.0 ± 0.0

**Table 3:** PERMANOVA results of parasitoid communities in ditches relative to month (June–August), year (2021–2023), farm (A–E), adjacent crop type, and the three transects sampled.

	Df	Sum of Sqs	R^2^	F	Pr (>F)
Month	2	2.717267	0.065	10.3544	0.001
Year	2	2.260914	0.054	8.6155	0.001
Farm	4	5.385900	0.128	10.2618	0.001
Crop	1	0.089933	0.002	0.6854	0.741
Transect	2	0.183463	0.004	0.6991	0.822
Residual	232	30.441351	0.725		

**Table 4:** Mean (±S.E.) rates of capture per card over 7 days and the rate of presence in traps for all families collected from agricultural ditches over the course of the study.

Family	Mean (±SE) Number Captured/Card/7 Days	Presence Rate	Family	Mean (±SE) Number Captured/Card/7 Days	Presence Rate
Mymaridae	13.5 ± 1.2	0.96	Eupelmidae s.l.	0.12 ± 0.027	0.09
Trichogrammatidae	10.0 ± 0.98	0.88	Eurytomidae	0.082 ± 0.020	0.07
Scelionidae	9.8 ± 0.62	0.99	Tiphiidae	0.066 ± 0.020	0.05
Figitidae	7.7 ± 0.61	0.92	Chalcididae	0.041 ± 0.014	0.04
Ceraphronidae	6.9 ± 0.60	0.92	Perilampidae	0.025 ± 0.010	0.02
Platygastridae	4.0 ± 0.36	0.76	Dryinidae	0.025 ± 0.014	0.02
Aphelinidae s.l.	3.3 ± 0.40	0.68	Sierolomorphidae	0.016 ± 0.010	0.01
Eulophidae	2.4 ± 0.16	0.75	Scoliidae	0.012 ± 0.0071	0.01
Encyrtidae	1.9 ± 0.26	0.54	Thynnidae	0.0082 ± 0.0058	0.01
Bethylidae	1.8 ± 0.34	0.34	Signiphoridae	0.0082 ± 0.0058	0.01
Pteromalidae s.l.	1.3 ± 0.12	0.61	Chrysididae	0.0082 ± 0.0058	0.01
Braconidae	1.2 ± 0.11	0.56	Torymidae	0.0082 ± 0.0058	0.01
Diapriidae	0.64 ± 0.071	0.39	Eucharitidae	0.0041 ± 0.0041	>0.01
Ichneumonidae	0.32 ± 0.061	0.16	Proctotrupidae	0.0041 ± 0.0041	>0.01
Megaspilidae	0.21 ± 0.056	0.12	Unk. family	0.14 ± 0.028	

**Table 5:** Mean (±S.E.) for abundance of the most common families collected from agricultural ditches over each month throughout the course of the study. Within each row, values followed by different letters are significantly different from each other at the α = 0.05 significance level (per Holm-adjusted Dunn tests).

Family	Number Captured/Card/7 Days (Mean ± SE)		
	June	July	August
Mymaridae	10.50 ± 1.16 a	9.20 ± 1.53 b	20.43 ± 2.82 c
Trichogrammatidae	7.32 ± 0.98 a	5.11 ± 0.74 b	17.29 ± 2.41 c
Scelionidae	9.84 ± 0.92 a	8.64 ± 0.70 a	10.95 ± 1.43 a
Figitidae	9.33 ± 1.11 a	5.83 ± 0.76 b	8.02 ± 1.22 ab
Ceraphronidae	7.01 ± 1.06 a	8.68 ± 1.32 a	5.08 ± 0.57 a
Platygastridae	3.91 ± 0.53 a	4.33 ± 0.73 a	3.83 ± 0.58 a
Aphelinidae s.l.	7.63 ± 1.08 a	1.62 ± 0.25 b	1.07 ± 0.14 b
Eulophidae	2.80 ± 0.31 a	2.05 ± 0.27 b	2.26 ± 0.26 ab
Encyrtidae	2.83 ± 0.71 a	1.73 ± 0.33 a	1.26 ± 0.21 a
Pteromalidae s.l.	1.86 ± 0.24 a	0.85 ± 0.13 a	1.38 ± 0.22 b
Braconidae	1.46 ± 0.23 a	0.81 ± 0.11 a	1.38 ± 0.22 a

**Table 6:** Mean (±S.E.) of the number captured per card per 7 days for common parasitoid families collected on each of the five farms from the three distances sampled (with 0 m representing samples within ditch vegetation). Within each family, values followed by different letters are significantly different from each other at the α = 0.05 significance level (per Holm-adjusted Dunn tests).

Family	Distance to Ditch	Number Captured/Card/7 Days (Mean ± SE)				
		Farm A	Farm B	Farm C	Farm D	Farm E
		Mean ± SE	Mean ± SE	Mean ± SE	Mean ± SE	Mean ± SE
Mymaridae	0 m	17.5 3± 4.45 a	17.13 ± 1.94 a	12.60 ± 2.30 a	9.26 ± 0.73 a	9.22 ± 1.36 a
	1.5 m	30.77 ± 5.73 ab	8.24 ± 1.01 abc	12.15 ± 2.47 ac	15.85 ± 1.75 b	3.78 ± 1.66 c
	9.1 m	20.91 ± 3.79 ab	11.55 ± 1.54 ab	19.15 ± 4.94 ab	19.25 ± 2.59 a	4.62 ± 1.32 b
Trichogrammatidae	0 m	14.91 ± 3.71 a	7.38 ± 0.57 a	2.60 ± 0.47 b	16.76 ± 1.75 c	6.75 ± 1.03 a
	1.5 m	1.56 ± 0.26 ab	1.17 ± 0.18 ac	0.54 ± 0.12 c	5.57 ± 0.73 d	1.57 ± 0.39 bd
	9.1 m	0.65 ± 0.13 a	1.32 ± 0.27 ab	0.62 ± 0.15 a	5.66 ± 0.91 c	1.36 ± 0.45 bc
Scelionidae	0 m	9.79 ± 2.13 a	7.74 ± 0.65 a	14.23 ± 1.24 b	9.76 ± 1.01 a	6.19 ± 0.62 a
	1.5 m	5.79 ± 0.67 a	4.85 ± 0.47 a	12.56 ± 1.66 b	7.20 ± 0.90 ab	4.22 ± 1.28 a
	9.1 m	2.19 ± 0.24 a	5.04 ± 0.64 b	9.28 ± 0.75 c	4.64 ± 0.62 b	4.09 ± 0.83 bc
Figitidae	0 m	9.89 ± 1.60 ab	5.74 ± 1.15 ab	5.40 ± 0.87 a	10.15 ± 1.48 b	6.75 ± 1.42 ab
	1.5 m	4.27 ± 0.86 ab	2.07 ± 0.31 a	4.56 ± 0.84 ab	7.39 ± 2.26 b	1.96 ± 0.81 ab
	9.1 m	3.17 ± 0.69 a	2.43 ± 0.32 a	2.74 ± 0.44 a	5.00 ± 0.97 a	1.74 ± 0.53 a
Ceraphronidae	0 m	2.91 ± 0.32 a	5.83 ± 0.76 b	16.44 ± 2.10 c	3.46 ± 0.47 ab	5.75 ± 0.87 ab
	1.5 m	2.81 ± 0.33 ab	3.96 ± 0.50 a	12.81 ± 1.66 c	2.35 ± 0.52 b	1.00 ± 0.29 b
	9.1 m	1.93 ± 0.28 a	4.06 ± 0.57 b	14.91 ± 2.26 c	1.81 ± 0.24 a	1.13 ± 0.35 a
Platygastridae	0 m	2.77 ± 0.59 ab	3.30 ± 0.44 c	1.81 ± 0.56 b	4.43 ± 0.65 c	10.25 ± 1.57 d
	1.5 m	0.25 ± 0.08 a	0.48 ± 0.13 a	0.37 ± 0.10 a	0.54 ± 0.10 a	1.80 ± 0.49 b
	9.1 m	0.15 ± 0.06 a	0.28 ± 0.08 a	0.09 ± 0.04 a	0.47 ± 0.16 a	3.42 ± 0.96 b
Aphelinidae s.l.	0 m	2.25 ± 0.52 ab	5.40 ± 1.08 c	3.00 ± 0.73 abc	3.98 ± 1.08 ac	0.94 ± 0.21 b
	1.5 m	0.71 ± 0.17 a	3.96 ± 0.89 bc	5.19 ± 1.17 b	2.46 ± 0.62 ac	1.57 ± 0.65 abc
	9.1 m	0.74 ± 0.19 a	5.23 ± 1.36 bc	5.60 ± 1.14 b	2.36 ± 0.65 ac	1.70 ± 0.74 bc
Eulophidae	0 m	1.70 ± 0.23 ab	3.47 ± 0.46 c	1.40 ± 0.23 a	3.04 ± 0.38 bc	2.00 ± 0.34 abc
	1.5 m	0.87 ± 0.13 a	0.54 ± 0.10 a	0.59 ± 0.13 a	0.63 ± 0.08 a	0.48 ± 0.19 a
	9.1 m	0.69 ± 0.12 a	0.55 ± 0.13 ab	0.17 ± 0.06 b	0.70 ± 0.14 a	0.34 ± 0.16 ab
Encyrtidae	0 m	0.70 ± 0.18 a	2.81 ± 0.79 b	2.85 ± 0.77 ab	0.83 ± 0.12 ab	2.72 ± 0.60 b
	1.5 m	0.13 ± 0.08 a	0.63 ± 0.15 b	1.13 ± 0.29 bc	0.35 ± 0.09 ab	1.61 ± 0.62 c
	9.1 m	0.11 ± 0.05 a	0.55 ± 0.14 a	0.53 ± 0.15 a	0.34 ± 0.08 a	3.47 ± 1.35 b
Pteromalidae s.l.	0 m	0.91 ± 0.21 a	1.94 ± 0.29 b	1.10 ± 0.16 ab	1.09 ± 0.17 ab	1.91 ± 0.52 ab
	1.5 m	0.42 ± 0.11 a	0.43 ± 0.12 a	1.09 ± 0.31 a	0.26 ± 0.07 a	0.33 ± 0.16 a
	9.1 m	0.85 ± 0.21 a	0.32 ± 0.08 a	0.28 ± 0.08 a	0.55 ± 0.17 a	0.62 ± 0.45 a
Braconidae	0 m	0.87 ± 0.21 a	0.91 ± 0.17 a	2.40 ± 0.37 b	0.63 ± 0.11 a	1.31 ± 0.26 ab
	1.5 m	0.90 ± 0.19 a	0.59 ± 0.10 a	2.28 ± 0.27 b	0.89 ± 0.16 a	0.91 ± 0.33 a
	9.1 m	0.56 ± 0.11 a	0.55 ± 0.12 a	1.92 ± 0.39 b	0.53 ± 0.12 a	0.70 ± 0.25 ab

## Data Availability

All the data are available within the Supplementary Materials.

## Supplementary Materials

The following supporting information can be downloaded at: https://www.mdpi.com/article/10.3390/insects16030246/s1, Table S1: Mean (±S.E.) ditch vegetation height around each transect, measured throughout 2021; Table S2: Dates of sample collection at each farm; Table S3: Pairwise PERMANOVA results for each pairwise comparison between farms; Table S4: Pairwise PERMANOVA results for each pairwise comparison between the three distances sampled; Table S5: PERMANOVA results for samples taken within fields adjacent to agricultural ditches, “Location” refers to the differences between the samples taken 1.5 m vs. 9.1 m away from the ditch habitat.
