# Supplementary material for: Family-Level Diversity of Hymenopteran Parasitoid Communities in Agricultural Drainage Ditches and Implications for Biological Control
Source: Insects. 2025 Feb 27;16(3):246. doi: 10.3390/insects16030246 (PMC11942648; doi:10.3390/insects16030246)
Supplement: Supplementary file 1 [file insects-16-00246-s001.zip › Supplemental Material Tables.pdf]

Supplementary Materials

Table S1. Mean (± S.E.) ditch vegetation height around each transect, measured throughout 2021.

**Farm**	**Vegetation Height (Mean ± SE, cm)**		
	**June**	**July**	**August**
**A**	**33.7 ± 1.2**	**51.5** ± 1.6	101.5 ± 3.7
B	68.9 ± 3.0	101.7 ± 4.2	106.8 ± 2.3
C	133.2 ± 2.7	110.1 ± 2.4	131.1 ± 1.8
D	40.6 ± 1.8	44.5 ± 1.9	84.1 ± 1.3
E	Not collected	90.7 ± 2.5	Not collected

Table S2. Dates of sample collection at each farm.

**Sample**		**Farm A**	**Farm B**	**Farm C**	**Farm D**	**Farm E**
Jun 2021	Set-up	9 June 2021	9 June 2021	15 June 2021	15 June 2021	—
	Collected	16 June 2021	16 June 2021	23 June 2021	23 June 2021	—
Jul 2021	Set-up	8 July 2021	7 July 2021	8 July 2021	6 July 2021	6 July 2021
	Collected	15 July 2021	July 2021	13 July 2021	13 July 2021	13 July 2021
Aug 2021	Set-up	12 August 2021	11 August 2021	12 August 2021	10 August 2021	10 August 2021
	Collected	19 August 2021	18 August 2021	19 August 2021	17 August 2021	17 August 2021
Jun 2022	Set-up	7 June 2022	7 June 2022	9 June 2022	9 June 2022	—
	Collected	14 June 2022	14 June 2022	16 June 2022	16 June 2022	—
Jul 2022	Set-up	14 July 2022	12 July 2022	14 July 2022	11 July 2022	11 July 2022
	Collected	21 July 2022	19 July 2022	21 July 2022	18 July 2022	18 July 2022
Aug 2022	Set-up	18 August 2022	16 August 2022	18 August 2022	15 August 2022	15 August 2022
	Collected	25 August 2022	23 August 2022	25 August 2022	22 August 2022	22 August 2022
Jun 2023	Set-up	7 June 2023	8 June 2023	7 June 2023	6 June 2023	6 June 2023
	Collected	14 June 2023	15 June 2023	16 June 2023	13 June 2023	13 June 2023
Jul 2023	Set-up	6 July 2023	5 July 2023	6 July 2023	5 July 2023	—
	Collected	13 July 2023	12 July 2023	13 July 2023	12 July 2023	—
Aug 2023	Set-up	3 August 2023	3 August 2023	2 August 2023	1 August 2023	1 August 2023
	Collected	10 August 2023	10 August 2023	9 August 2023	8 August 2023	8 August 2023

Table S3. Pairwise PERMANOVA results for each pairwise comparison between farms.

Comparison	Farm df	Residual df	Farm SumOfSqs	Residual SumOfSqs	Total SumOfSqs	R^2	F	Pr(>F)
A vs. B	1	104	0.157440849	3.918319684	4.075760533	0.038628582	4.178793368	0.003
A vs. C	1	103	0.669311201	5.527821583	6.197132785	0.108003366	12.47128778	0.001
A vs. D	1	105	0.147144188	4.220272626	4.367416814	0.033691354	3.660934047	0.003
A vs. E	1	83	0.206656117	3.497327047	3.703983164	0.055792942	4.904447746	0.001
B vs. C	1	103	0.602397572	4.617982188	5.22037976	0.115393439	13.43594397	0.001
B vs. D	1	105	0.05548858	3.310433231	3.365921811	0.016485404	1.75998141	0.141
B vs. E	1	83	0.121832875	2.587487652	2.709320527	0.044968055	3.908087698	0.007
C vs. D	1	104	0.701380218	4.91993513	5.621315348	0.124771548	14.82611878	0.001
C vs. E	1	82	0.491214163	4.196989551	4.688203714	0.104776625	9.597250817	0.001
D vs. E	1	84	0.143797745	2.889440594	3.033238339	0.047407335	4.180397633	0.002

Table S4. Pairwise PERMANOVA results for each pairwise comparison between the three distances sampled.

Comparison	Location df	Residual df	Location SumOfSqs	Residual SumOfSqs	Total SumOfSqs	R2	F	Pr(>F)
Ditch vs. Field (1.5m)	1	488	1.667727791	30.83698176	32.50470955	0.051307266	26.39204992	0.001
Ditch vs. Field (9.1m)	1	490	3.157334594	31.63244502	34.78977961	0.090754659	48.90845302	0.001
Field (1.5m vs. 9.1m)	1	492	0.356482556	39.93956705	40.2960496	0.008846588	4.391370024	0.001

Table S5. PERMANOVA results for samples taken within fields adjacent to agricultural ditches, "Location" refers to the differences between the samples taken 1.5m vs. 9.1m away from the ditch habitat.

	**Df**	**Sum of Sqs**	**R^2**	**F**	**Pr(>F)**
Year	2	4.577229598	0.044237858	15.62322723	0.001
Month	2	12.08866303	0.116834111	41.26162463	0.001
Farm	4	12.24009974	0.118297712	20.88925795	0.001
Crop	1	1.529690811	0.014784105	10.44243319	0.001
Transect	2	0.135699716	0.001311506	0.463177004	0.98
Location	1	0.519610415	0.005021914	3.547120112	0.001
Residual	481	70.46071224	0.680986366		
